# Clinical Manifestations of Infections with the Omicron Sub-Lineages BA.1, BA.2, and BA.5: A Retrospective Follow-Up Analysis of Public Health Data from Mecklenburg-Western Pomerania, Germany

**DOI:** 10.3390/v16030454

**Published:** 2024-03-15

**Authors:** Katja Verena Goller, Janine Ziemann, Christian Kohler, Karsten Becker, Nils-Olaf Hübner, on behalf of the CoMV-Gen Study Group

**Affiliations:** 1 Central Unit for Infection Prevention and Control and Institute for Hygiene and Environmental Medicine, University Medicine Greifswald, 17475 Greifswald, Germany; janine.ziemann@med.uni-greifswald.de; 2 Friedrich-Loeffler-Institute of Medical Microbiology, University Medicine Greifswald, 17475 Greifswald, Germany; christian.kohler@med.uni-greifswald.de (C.K.); karsten.becker@med.uni-greifswald.de (K.B.)

**Keywords:** SARS-CoV-2, Omicron, BA.1, BA.2, BA.5, symptoms, COVID-19, surveillance, Mecklenburg-Western Pomerania, Germany, pandemic

## Abstract

The Omicron variants BA.1, BA.2, and BA.5 caused several waves of SARS-CoV-2 in Germany in 2022. In this comparative study, public health data on SARS-CoV-2 infections from Mecklenburg-Western Pomerania, Germany, between January and October 2022 were examined retrospectively using Pearson’s chi-squared tests and Fisher’s exact tests for testing for statistical significance. Compared to BA.5 infections, BA.1 and BA.2 infections affected younger individuals aged up to 19 years significantly more often, whereas BA.5 infections occurred significantly more frequently in patients between 40 and 59 years of age when compared to BA.1 and BA.2. Infections with all three variants predominantly caused flu-like symptoms; nevertheless, there were significant differences between the reported symptoms of BA.1, BA.2, and BA.5 infections. Especially, the symptoms of ‘fever’, ‘severe feeling of sickness’, ‘loss of taste’, and ‘loss of smell’ were significantly more often present in patients with BA.5 infections compared to BA.1 and BA.2 cases. Additionally, BA.2 and BA.5 cases reported significantly more often the symptoms of ‘runny nose’ and ‘cough’ than BA.1-infected cases. Our findings indicate remarkable differences in the clinical presentations among the sub-lineages, especially in BA.5 infections. Furthermore, the study demonstrates a powerful tool to link epidemiological data with genetic data in order to investigate their potential impact on public health.

## 1. Introduction

As of 5 May 2023, the World Health Organization (WHO) declared, due to the decreasing numbers of infections and the higher overall immunity among the public, that the Severe Acute Respiratory Syndrome Virus 2 (SARS-CoV-2), causing the disease COVID-19, remains an ongoing public health issue but does not constitute a public health emergency of international concern (PHEIC) anymore [1]. However, the ongoing pandemic caused by SARS-CoV-2 continues to affect people worldwide. The virus leads to a range of symptoms in humans, with some being severe [2,3]. Due to its high mutation rate, common for RNA viruses, new variants of the virus constantly emerged during the pandemic. Some of these variants had significantly different characteristics and triggered new waves of the pandemic. One such variant is B.1.1.529 (Omicron) and its sub-lineages as well as recombinant lineages, which have been the dominant strains globally from late 2021 to 2023. Omicron was first identified in South Africa and was classified as a ‘variant of concern’ (VOC) on 26 November 2021 [4,5] and quickly gave rise to multiple sub-lineages. In Germany, the BA.1 variant replaced the lineage B.1.617.2 ‘Delta’ as the predominant variant during the 52nd calendar week of 2021, accounting for 53% of cases [3]. Early in 2022, SARS-CoV-2 cases increased steeply, leading to the emergence of the fifth wave, known as the ‘first Omicron wave’, in Germany [3]. After two months of BA.1 dominance early in 2022, the sub-lineage B.1.1.529.2 (BA.2) took over as the main variant in Germany, constituting 63.1% of BA.2 cases [3], and case numbers continued to rise. Thus, the BA.2 variant quickly replaced BA.1 by the end of February 2022 and was the dominant variant until June 2022 in Germany. However, subsequently, another sub-lineage, BA.5, was firstly detected in Germany by the end of February, and a rapid increase in this sub-lineage was observed, in which BA.5 superseded BA.2 by the end of June 2022 [6].

The transmissibility and immune escape of SARS-CoV-2 variants are caused by specific mutations that can alter the virus behavior and thus influence the spread, clinical severity, and clinical presentation of the virus [7,8,9,10]. Compared to other pre-Omicron variants, such as ‘Delta’, the Omicron BA.1 and BA.2 sub-lineages showed a higher transmissibility but less severe symptoms [11,12,13]. For instance, the median duration of acute symptomatic illness was eight days in cases caused by the BA.1 variant in comparison to five days in cases caused by the Delta variant [11]. Differences between BA.5 and BA.2 could also be observed in terms of BA.5 having a higher transmissibility and being more pathogenic than BA.2. Additionally, the genetic differences among the Omicron subvariants could also imply potential differences in clinical presentations, probably caused by different viral loads in the respiratory tract [14,15]. However, despite the data on mortality [16], direct comparisons of the symptoms caused by BA.1 and BA.2 infections are rare, but suggest somewhat milder symptoms in BA.1 infections [17]. In a previous study, we investigated differences in clinical presentations between BA.2- and BA.5-infected cases [18]. This study was based on public health data from a short time period between the end of April 2022 and mid-July 2022. This reflected the time-frame when BA.2 was replaced by BA.5. Significant differences between the symptoms reported from BA.2 and BA.5 cases could be observed, but no higher pathogenicity of BA.5 compared to BA.2 could be confirmed [18].

In order to test whether our approach to use public health data for analyzing differences in clinical presentations could be extended and to increase the sample size and retrospectively compare the sub-lineages BA.1, BA.2, and BA.5, we aimed to conduct this follow-up study covering a larger time-frame and including an extended dataset based on officially reported data between January 2022 and October 2022 from the public health authorities of the federal state of Mecklenburg-Western Pomerania, Germany.

## 2. Materials and Methods

Official surveillance data from the public health authorities of Mecklenburg-Western Pomerania were obtained for this study. Epidemiological data were collected either through structured self-report questionnaires or through interviews with patients by public health officials and recorded in SORMAS (Surveillance Outbreak Response Management and Analysis System [19]). The dataset for statistical analyses was extracted, anonymized, and retrospectively analyzed. As previously described, CoMV-Gen was commissioned by the government of the federal state of Mecklenburg-Western Pomerania, Germany, and funded by the Ministry of Social Affairs, Health and Sports for the SARS-CoV-2 surveillance in Mecklenburg-Western Pomerania, including the acquisition of genetic information from, e.g., whole-genome-sequencing analyses in collaboration with local diagnostic laboratories [18]. Approval for ethical considerations was obtained from the ethics committee at the University of Greifswald, Germany (BB 125/21).

Data were gathered from individuals identified as having contracted the Omicron variant. All cases that could be clearly assigned to either BA.1, BA.2, and BA.5 or one of their sub-lineages based on the SORMAS entries were included, while other Omicron variants occurring during the study period were excluded from further analyses. 

Data from the 1st ISO (International Organization for Standardization) calendar week in January 2022 (first case: 5 January 2022) to the 41st ISO calendar week in October 2022 (last case: 11 October 2022) from Mecklenburg-Western Pomerania were used. To explore the distribution of infections among different age groups within the positive cases, individuals were categorized into five age groups spanning 20-year intervals: 0–19, 20–39, 40–59, 60–79, and 80 years old and above. For comparing the symptoms among the three sub-lineages, only data from cases that reported symptoms were used. The 21 COVID-19-relevant symptoms were selected based on the standardized and cross-sectional German Corona Consensus Dataset (GECCO) for research [20].

SPSS 28.0.1.0 (IBM SPSS Statistics for Windows, Version 28.0.1.0 Armonk, NY, USA: IBM Corp.) was used for the statistical analyses. Pearson’s chi-squared tests and Fisher’s exact tests were used for testing for statistical significance and the 95% confidence intervals (CI95) were determined. A statistical significance was considered as present if the *p*-value was less than 0.05 (*p* < 0.05). For statistical analyses of the epidemiological characteristics as well as for the comparative analyses, Pearson’s chi-squared tests with Bonferroni adjustment were conducted in order to examine whether there were overall significant differences among all lineages. In the case of significant results, as a second step, Pearson’s chi-squared tests with Bonferroni adjustments were conducted to compare each variant against each other. In order to investigate differences between the age groups and the three lineages, Fisher’s exact tests were used. 

## 3. Results

**Figure 1:**
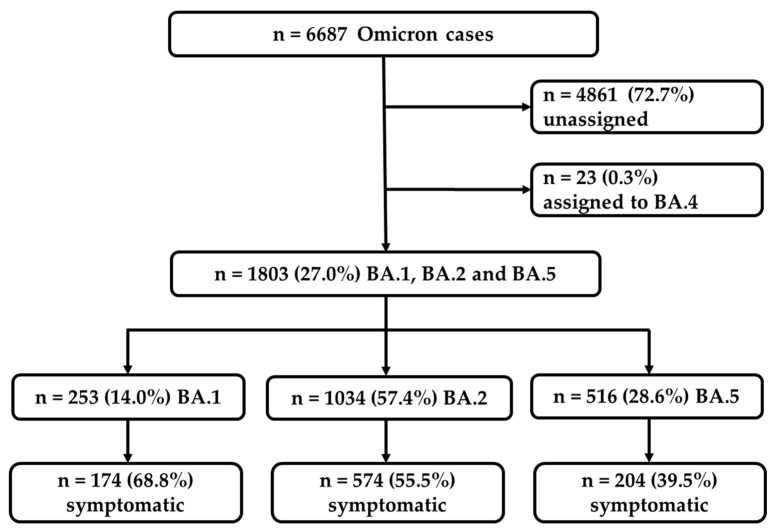
Flowchart representing the selection of the confirmed BA.1, BA.2, and BA.5 Omicron cases used for the further analyses of symptomatic cases.

During the study period, spanning from the 1st ISO calendar week of January to the 41st week of October 2022, a total of 6687 cases of the Omicron variants were confirmed (Figure 1). The majority of these cases, 4884 in number (73.0%), were either not assigned to any specific Omicron sub-lineage (4861 cases) or were assigned to the sub-lineage BA.4 (23 cases). These particular cases were excluded from further analyses (Figure 1). Out of the remaining 1803 cases, 253 (14.0%) cases were identified as belonging to the sub-lineage BA.1, 1034 (57.4%) cases belonged to BA.2, and 516 (28.6%) were assigned to BA.5. Among these, a total of 952 cases (52.8%) were reported as being symptomatic and were used for the purpose of further comparative analyses (Figure 1). The symptomatic cases consisted of 174 BA.1 cases (68.8%), 574 BA.2 cases (55.5%), and 204 BA.5 cases (39.5%).

**Figure 2:**
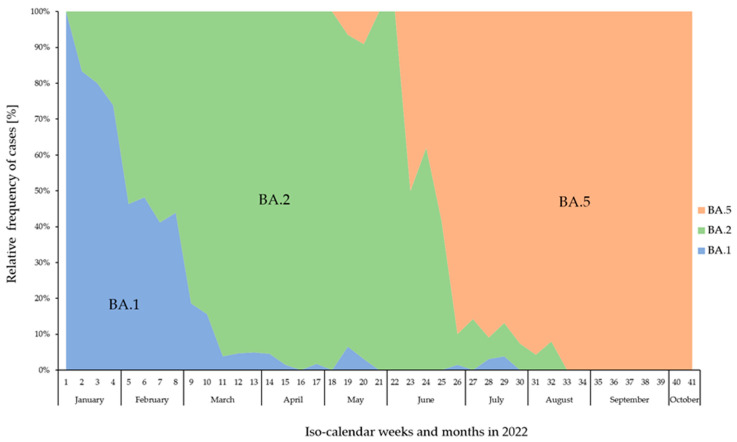
Relative frequency (%) of confirmed BA.1 (blue), BA.2 (green), and BA.5 (orange) cases across the calendar weeks from January to October 2022 in Mecklenburg-Western Pomerania, Germany.

The sub-lineage BA.1 was the most prominent variant until the end of January 2022. However, by the end of January, an increase in the proportion of the sub-lineage BA.2 could already be observed, which almost completely displaced BA.1 by the beginning of March 2022 and became the dominant variant until the beginning of June. By the end of May 2022, the sub-lineage BA.5 appeared and increased rapidly and supplanted BA.2 almost completely by the end of June 2022 (Figure 2). 

The epidemiological characteristics of the BA.1, BA.2, and BA.5 cases are summarized in Table 1. No significant differences in the proportion of infected male and female cases among BA.1, BA.2, and BA.5 as well as in the survival status of infected individuals could be observed (Table 1).

**Figure 3:**
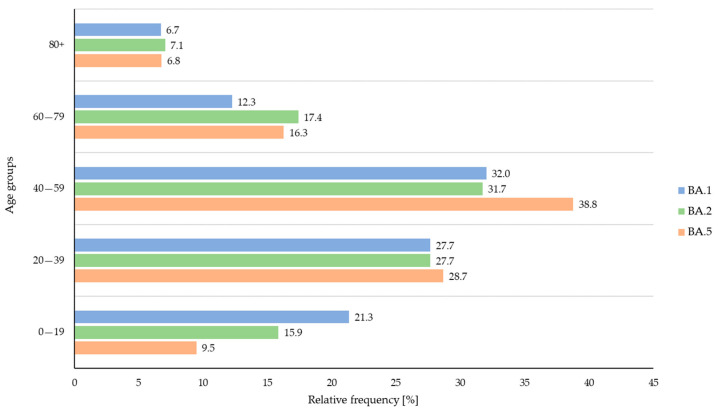
Relative frequency (%) of confirmed BA.1 (blue), BA.2 (green), and BA.5 (orange) cases across the age groups during the study period.

A total of 16 deaths were registered during the study period (0.9% of all Omicron cases, amongst them 6 females and 10 males between 60 and 94 years of age). There was no significant difference in the deceased cases among BA.1-, BA.2-, and BA.5-infected individuals (two BA.1 cases: 0.8%; ten BA.2 cases: 1.0%; and four BA.5 cases: 0.8%). Strong significant differences could be observed when comparing the number of reported symptoms among all three variants using Pearson’s chi-squared tests (*p* < 0.001). When comparing each variant against each other, the statistical analyses revealed that BA.1 cases reported symptoms more frequently than BA.2 and BA.5 cases (*p* < 0.001) and BA.2 cases reported symptoms more frequently than BA.5 cases (*p* < 0.001). In terms of vaccination status, BA.5 cases appeared to be more frequently vaccinated than BA.1 and BA.2 cases (*p* < 0.001) and BA.2 cases were more often vaccinated than BA.1 cases (*p* = 0.003). However, data on vaccination status were only provided for a low proportion of cases overall. Similarly, this also accounted for the re-infection data: only small numbers of re-infections were reported, but there was an overall significant difference among the variants (*p* < 0.012) when the unknown data were excluded from the analyses. The direct comparison revealed that BA.5 cases tended to suffer more frequently from re-infections than BA.2 cases (*p* = 0.019) and BA.1 cases (*p* = 0.015). In terms of age, Fisher’s exact tests were performed to examine differences among all age categories and the three variants (Figure 3). 

Overall, BA.1 and BA.2 infections were more frequently observed in young individuals (age group 1: 0–19 years of age; BA.1 vs. BA.2, *p* = 0.037; BA.1 vs. BA.5, *p* < 0.001; and BA.2 vs. BA.5, *p* = 0.006). In contrast, individuals infected with BA.5 belonged more frequently to age group 3 (40–59 years of age) when compared to BA.1 and BA.2 cases (*p* = 0.006).

In total, 174 BA.1, 574 BA.2, and 204 BA.5 cases showed one or more symptoms (Table 2). The 21 listed symptoms correspond to the mainly observed key symptoms. Overall, significant differences among all three sub-lineages by conducting Pearson’s chi-squared tests could be observed in the symptoms of ‘runny nose’ (*p* = 0.032), ‘cough’ (*p* < 0.001), ‘sore throat/pharyngitis’ (*p* = 0.015), ‘fever’ (*p* < 0.001), ‘severe feeling of sickness’ (*p* < 0.001), and ‘loss of smell’ (*p* = 0.015) (Table 2). 

**Figure 4:**
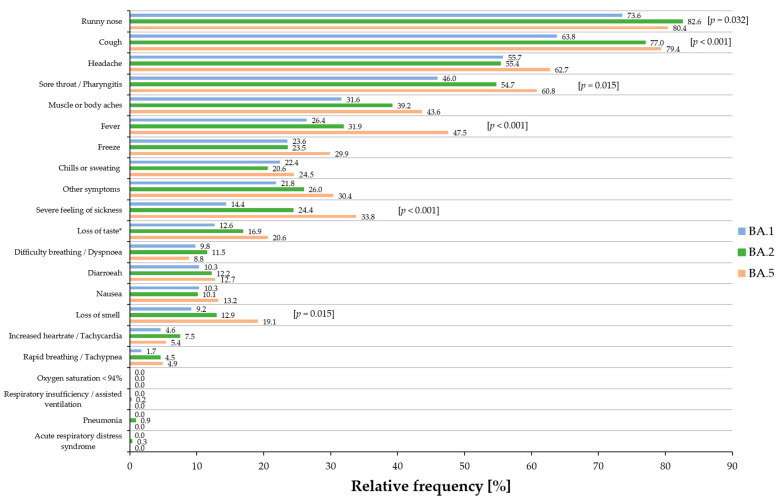
Relative frequency of the most prominent 21 symptoms of symptomatic BA.1 (blue), BA.2 (green), and BA.5 (orange) cases. Overall *p*-values are presented in squared brackets for significant results. * a significant result (*p* = 0.040) was obtained for ‘loss of taste’ between BA.1 and BA.5.

Additionally, the relative frequencies of the main symptoms for all sub-lineages are shown in Figure 4. When comparing each variant against each other by Pearson’s chi-squared tests, in detail, BA.2 and BA.5 cases reported significantly more often the symptom of ‘runny nose’ than BA.1-infected individuals (*p* = 0.009), but no significant difference could be observed between BA.2 and BA.5 cases. The same applied to the symptom of ‘cough’: no significant difference could be observed between BA.2 and BA.5 cases, but BA.1 cases reported this symptom less frequently than BA.2 and BA.5 cases (*p* < 0.001 respectively). Regarding the symptom of ‘sore throat/pharyngitis’, BA.1 cases reported this symptom less frequently than BA.2 cases (*p* = 0.043) and BA.5 cases (*p* = 0.004). No significant difference could be observed between BA.5 and BA.2 cases. BA.5 cases reported most frequently on the symptom of ‘fever’. When comparing this symptom to that of BA.1 and BA.2 cases, a high statistical significance could be observed (BA.1 vs. BA.5 and BA.2. vs. BA.5, *p* < 0.001, respectively), while no significant difference could be observed between BA.1 and BA.2 cases. The symptom of ‘severe feeling of sickness’ was most frequently reported by BA.5 cases when compared to BA.1 (*p* < 0.001) and BA.2 cases (*p* = 0.009), but also BA.2 cases reported this symptom more frequently than BA.1 cases (*p* = 0.005). Individuals infected with BA.5 suffered more frequently from ‘loss of taste’ than BA.1-infected cases (*p* = 0.040), but no significant difference could be observed between BA.1 and BA.2 cases and between BA.2 and BA.5 cases. A similar situation occurred for the symptom of ‘loss of smell’, in which BA.5 cases reported this symptom more frequently than BA.1- and BA.2-infected cases (BA.1 vs. BA.5, *p* =0.006; BA.2 vs. BA.5, *p* = 0.030; BA.1 vs. BA.2, not significant). When comparing each sub-lineage against each other, a significant difference could be observed between BA.1 and BA.5, in which the symptom of ‘muscle or body aches’ was reported more frequently by BA.5 cases than BA.1 cases (*p* = 0.016), but a significant difference could be observed between BA.1 and BA.2 cases and between BA.2 and BA.5 cases.

Severe symptoms, such as ‘acute respiratory distress syndrome’, ‘respiratory insufficiency/assisted breathing’, ‘pneumonia’, and ‘oxygen saturation < 94%’, were reported rarely or not at all for all three sub-lineages.

## 4. Discussion

In this follow-up study, we examined the clinical manifestations of SARS-CoV-2 infections with the sub-lineages BA.1, BA.2, and BA.5, including their subvariants, based on official data provided by the public health authorities of the federal state of Mecklenburg-Western Pomerania, Germany. Although the diminished impact of various Omicron variants on disease severity has been well documented [21,22,23], data on the detailed clinical presentation of these variants are still sparse. To the best of our knowledge, we report for the first time the differences in the symptoms reported by individuals infected either with BA.1, BA.2, or BA.5 based on public health data, as the most comprehensive source of information available, notwithstanding some limitations mentioned below.

In our investigation, the predominant symptoms reported for all three sub-lineages primarily resulted in respiratory symptoms, and with only a minority of cases in the sub-lineages leading to a more severe course of infection. Nevertheless, a notable proportion of cases—twelve percent of BA.2 cases, approximately ten percent of BA.1 cases, and nine percent of BA.5 cases—reported ‘breathing difficulties’ (dyspnea). Other symptoms typically linked to severe illness, such as ‘pneumonia’, ‘acute respiratory distress syndrome’, or ‘respiratory insufficiency’, were only very rarely or even not at all reported in all cases. 

Our data show that BA.1 cases reported symptoms more frequently than BA.2 and BA.5 cases, and BA.2 cases reported even more frequently on being symptomatic than BA.5 cases. BA.2 has been reported to cause more severe infections and more symptoms compared to BA.1 [3,9,13]. Another study has shown that the symptoms of all sub-lineages were similar and focused on respiratory insufficiencies, but BA.2 may cause more and even more severe symptoms than BA.1 infections [17]. Compared to Delta and other VOC infections, Omicron infections have been reported to be less severe [11,12,13], and hospitalization rates, the need for intensive care, and mortality rates were much lower [11]. However, among the symptomatic cases in our study, the symptoms of ‘fever’, ‘severe feeling of sickness’, ‘sore throat/pharyngitis’, ‘loss of taste’, and ‘loss of smell’ were more frequently reported by BA.5 cases than BA.1 and BA.2 cases; however, ‘loss of smell’ was generally less frequently reported in Omicron infections than in Delta infections [11].

The symptoms of ‘runny nose’ and ‘cough’ occurred significantly more frequently in BA.2- and BA.5-infected individuals than in BA.1 cases. One possible explanation for this could be that there is evidence that patients infected with the BA.2 and BA.5 sub-lineages have a higher upper respiratory viral load compared to patients infected with BA.1 [14,15]. However, in accordance with the results of Whitaker et al. [24], respiratory symptoms were the most prominent in all three sub-lineages. 

In previous waves, including the Delta variant and those preceding Omicron, a significant number of individuals were not vaccinated. However, vaccination, particularly booster shots, plays a crucial role in providing protection against severe Omicron infections and the need for hospitalization [25,26,27,28]. Nevertheless, the vaccination data within our study are limited, given the relatively high number of unreported cases. This may be attributed to incomplete responses to the questionnaires from affected individuals, preventing us from drawing additional conclusions. While our data do not permit the differentiation of symptomatic cases between vaccinated and unvaccinated individuals, other studies have indicated a higher likelihood of infection among the unvaccinated [9,26]. Our findings underscore the need for additional information regarding the quantity and timing of vaccinations.

Our study has certain limitations that warrant consideration in interpreting the findings. Firstly, the dataset relied on manual entries made by public health authorities in SORMAS [19], which in turn are based on self-reports through questionnaires completed by infected individuals or by interviews with the affected person or their relatives. During the peak of the fifth wave, public health authorities faced substantial workloads that might have impacted the quality of data in the manual entries. Additionally, only data that allowed discrimination between the lineages BA.1, BA.2 and BA.5 within the study period were used. Prior to January 2022, public health authorities predominantly reported only ‘Omicron’, making it impossible to distinguish between the three lineages and to include additional data from BA.1 cases in the epidemiological dataset. While SORMAS offered to record information about ‘vaccination’, ‘re-infection’, and ‘status’, only few entries were made by the public health staff in this dataset. After October 2022, the public health authorities discontinued using the SORMAS database, resulting in the unavailability of epidemiological data pertaining to BA.5 cases for further investigations.

Furthermore, the reported severity of symptoms was subject to the patients’ subjective interpretation and may not precisely reflect their actual condition. Additionally, assigning symptoms to specific categories in the provided response options can be challenging: for instance, ‘muscle or body aches’ might also be reported in the ‘severe feeling of sickness’ category, and the symptom of ‘freeze’ might also be included in the ‘chills or sweating’ category. The recording of symptoms was highly reliant on the patient’s physical condition at the time of questioning and their willingness to provide information. This could result, for instance, in the duplication of certain symptoms and the introduction of systematic errors. Even when patient interviews or self-reporting were obligatory, there was no consistent follow-up on hospitalized cases, potentially resulting in the underreporting of severe cases. It is important to note, however, that these limitations are applicable to all three variants in our study. Secondly, in this investigation, descendants from the parent lineages BA.1, BA.2, and BA.5 were grouped together as BA.1, BA.2, and BA.5, preventing an examination of symptom variation at the descendant level due to the limited sample size. For instance, the diversity among the sub-lineages of the BA.2 lineage itself may contribute to variations in the immunologic context [29,30]. 

Throughout the study period, the genetic diversity of the sub-lineages exhibited a steady increase, although specific details are not presented in this paper. Distinct mutations in the Omicron genome have the potential to alter the behavior and characteristics of the virus, consequently impacting its spread and affecting patient outcomes [8,9,10,31]. For example, the BA.2 descendant BA.2.12.1 has developed a neutralization escape mechanism affecting both vaccinated individuals and those infected with BA.1 or BA.2, potentially leading to an increased infection rate and the manifestation of more symptoms [32]. 

The omicron variant BA.2 was first replaced by BA.5 in early June 2022, but a re-emergence of BA.2 was observed in early November 2022. However, a recombinant variant (XBB.1 and its descendants), characterized by a recombination of two BA.2 sub-lineages, BJ.1 (alias for B.1.1.529.2.10.1.1) and BM.1.1.1 (alias of B.1.1.529.2.75.3.1.1.1), was increasingly detected internationally and nationally as well as in Mecklenburg-Western Pomerania from late January 2023 onwards. Subsequently, it displaced both the original BA.2 variant and its sub-lineages and the BA.5 variant and its sub-lineages. The reappearance of BA.2 in November 2022, coupled with the rapid emergence of the BA.2-recombinant variant XBB.1 and its subvariants, underscores the importance of monitoring previous variants and studying their characteristics, even if it initially appears that they have already been supplanted.

## 5. Conclusions

Comprehensive analyses of epidemiological data related to circulating SARS-CoV-2 variants, including their clinical manifestations, are imperative for evaluating the present scenario and efficiently controlling the transmission of SARS-CoV-2. This approach enables the implementation or expansion of tailored protective measures. Identifying new variants or sub-lineages of the SARS-CoV-2 virus is crucial to understanding their potential impact on the population’s threat level. Our examination of the Omicron subvariants BA.1, BA.2, and BA.5 in Mecklenburg-Western Pomerania, Germany, utilizing data from public health authorities, has unveiled distinct differences in symptoms. All three sub-lineages pre-dominantly exhibited flu-like symptoms and a more or less mild course of infection. Nevertheless, BA.5 infections demonstrated a tendency to cause infections with more severe symptoms and had the capacity to induce a greater severity compared to BA.1 and BA.2. The re-emergence of BA.2 in 2022 as well as the rapid occurrence of the BA.2-recombinant variant XBB show that it is still necessary to monitor already forgotten variants. Additionally, our study shows the potential of public health data to describe and monitor clinical manifestations of different variants—but their current limitations, too. Therefore, our study underscores the potential for enhancements in Germany’s public health authority data as well as their availability for scientific analysis. We therefore advocate for more specific information on circulating variants to be available early on and integrated with epidemiological data. Utilizing surveillance systems for closely monitoring the evolution and clinical presentations of new variants is essential for scientific-based public health responses.

## Figures and Tables

**Table 1:** Epidemiological characteristics and analyses of the information from all recorded BA.1, BA.2, and BA.5 cases. Unknown cases were excluded from statistical analyses and are presented in italics.

Characteristics		BA.1		BA.2		BA.5		*p*-Value ^1^
		*n*	%	*n*	%	*n*	%	
Total	Cases	253	14.0	1.034	57.4	516	28.6	-
Sex	Female	125	49.4	537	51.9	285	55.2	0.247
	Male	127	50.2	491	47.5	231	44.8	
	*Unknown*	*1*	*0.4*	*6*	*0.6*	*0*	*0.0*	
Age groups	0–19	54	21.3	164	15.9	49	9.5	**<0.001**
	20–39	70	27.7	286	27.7	148	28.7	
	40–59	81	32.0	328	31.7	200	38.8	
	60–79	31	12.3	180	17.4	84	16.3	
	80+	17	6.7	73	7.1	35	6.8	
	*Unknown*	*0*	*0.0*	*3*	*0.3*	*0*	*0.0*	
Symptoms	Yes	174	68.8	574	55.5	204	39.5	**<0.001**
	No	79	31.2	460	44.5	312	60.5	
	*Unknown*	*0*	*0.0*	*0*	*0.0*	*0*	*0.0*	
Vaccination	Yes	96	37.9	354	34.2	164	31.8	**<0.001**
	No	41	16.2	79	7.6	10	1.9	
	*Unknown*	*116*	*45.8*	*601*	*58.1*	*342*	*66.3*	
Re-infection	Yes	2	0.8	10	1.0	12	2.3	**0.012**
	No	116	45.8	303	29.3	133	25.8	
	*Unknown*	*135*	*53.4*	*721*	*69.7*	*371*	*71.9*	
Status	Vital	197	77.9	588	56.9	94	18.2	0.145
	Deceased	2	0.8	10	1.0	4	0.8	
	*Unknown*	*54*	*21.3*	*436*	*42.2*	*418*	*81.0*	

^1^ Significant overall *p*-values are shown from Pearson’s chi squared statistics (in bold).

**Table 2:** Overall differences between the 21 key symptoms reported from symptomatic BA.1, BA.2, and BA.5 cases (CI, confidence interval (CI 95% [lower–upper]); Positive, positively reported; n.r., not reported.).

Symptom	Status	BA.1		BA.2		BA.5		*p*-Value ^1^
		*n* (%)	CI 95 [%]	*n* (%)	CI 95 [%]	*n* (%)	CI 95 [%]	
Runny nose	Positive	128 (73.6%)	[66.7–79.7]	474 (82.6%)	[79.3–85.5]	164 (80.4%)	[74.5–85.4]	**0.032**
	n.r.	46 (26.4%)	[20.3–33.3]	100 (17.4%)	[14.5–20.7]	40 (19.6%)	[14.6–25.5]	
Cough	Positive	111 (63.8%)	[56.5–70.7]	442 (77%)	[73.4–80.3]	162 (79.4%)	[73.5–84.5]	**<0.001**
	n.r.	63 (36.2%)	[29.3–43.5]	132 (23%)	[19.7–26.6]	42 (20.6%)	[15.5–26.5]	
Headache	Positive	97 (55.7%)	[48.3–63.0]	318 (55.4%)	[51.3–59.4]	128 (62.7%)	[56.0–69.2]	0.177
	n.r.	77 (44.3%)	[37.0–51.7]	256 (44.6%)	[40.6–48.7]	76 (37.3%)	[30.8–44.0]	
Sore throat/pharyngitis	Positive	80 (46%)	[38.7–53.4]	314 (54.7%)	[50.6–58.7]	124 (60.8%)	[54.0–67.3]	**0.015**
	n.r.	94 (54%)	[46.6–61.3]	260 (45.3%)	[41.3–49.4]	80 (39.2%)	[32.7–46.0]	
Muscle or body aches	Positive	55 (31.6%)	[25.0–38.8]	225 (39.2%)	[35.3–43.2]	89 (43.6%)	[37.0–50.5]	0.054
	n.r.	119 (68.4%)	[61.2–75.0]	349 (60.8%)	[56.8–64.7]	115 (56.4%)	[49.5–63.0]	
Fever	Positive	46 (26.4%)	[20.3–33.3]	183 (31.9%)	[28.2–35.8]	97 (47.5%)	[40.8–54.4]	**<0.001**
	n.r.	128 (73.6%)	[66.7–79.7]	391 (68.1%)	[64.2–71.8]	107 (52.5%)	[45.6–59.2]	
Other symptoms	Positive	38 (21.8%)	[16.2–28.4]	149 (26%)	[22.5–29.7]	62 (30.4%)	[24.4–36.9]	0.166
	n.r.	136 (78.2%)	[71.6–83.8]	425 (74%)	[70.3–77.5]	142 (69.6%)	[63.1–75.6]	
Severe feeling of sickness	Positive	25 (14.4%)	[9.8–20.2]	140 (24.4%)	[21.0–28.0]	69 (33.8%)	[27.6–40.5]	**<0.001**
	n.r.	149 (85.6%)	[79.8–90.2]	434 (75.6%)	[72.0–79.0]	135 (66.2%)	[59.5–72.4]	
Freeze	Positive	41 (23.6%)	[17.7–30.3]	135 (23.5%)	[20.2–27.1]	61 (29.9%)	[23.9–36.4]	0.175
	n.r.	133 (76.4%)	[69.7–82.3]	439 (76.5%)	[72.9–79.8]	143 (70.1%)	[63.6–76.1]	
Chills or sweating	Positive	39 (22.4%)	[16.7–29]	118 (20.6%)	[17.4–24]	50 (24.5%)	[19–30.7]	0.487
	n.r.	135 (77.6%)	[71.0–83.3]	456 (79.4%)	[76.0–82.6]	154 (75.5%)	[69.3–81.0]	
Loss of taste	Positive	22 (12.6%)	[8.3–18.2]	97 (16.9%)	[14.0–20.1]	42 (20.6%)	[15.5–26.5]	0.121
	n.r.	152 (87.4%)	[81.8–91.7]	477 (83.1%)	[79.9–86]	162 (79.4%)	[73.5–84.5]	
Loss of smell	Positive	16 (9.2%)	[5.6–14.2]	74 (12.9%)	[10.3–15.8]	39 (19.1%)	[14.2–24.9]	**0.015**
	n.r.	158 (90.8%)	[85.8–94.4]	500 (87.1%)	[84.2–89.7]	165 (80.9%)	[75.1–85.8]	
Diarrhea	Positive	18 (10.3%)	[6.5–15.5]	70 (12.2%)	[9.7–15.1]	26 (12.7%)	[8.7–17.8]	0.748
	n.r.	156 (89.7%)	[84.5–93.5]	504 (87.8%)	[84.9–90.3]	178 (87.3%)	[82.2–91.3]	
Breathing difficulties/dyspnea	Positive	17 (9.8%)	[6.0–14.8]	66 (11.5%)	[9.1–14.3]	18 (8.8%)	[5.5–13.3]	0.524
	n.r.	157 (90.2%)	[85.2–94]	508 (88.5%)	[85.7–90.9]	186 (91.2%)	[86.7–94.5]	
Nausea	Positive	18 (10.3%)	[6.5–15.5]	58 (10.1%)	[7.8–12.8]	27 (13.2%)	[9.1–18.4]	0.454
	n.r.	156 (89.7%)	[84.5–93.5]	516 (89.9%)	[87.2–92.2]	177 (86.8%)	[81.6–90.9]	
Increased heartrate/tachycardia	Positive	8 (4.6%)	[2.2–8.5]	43 (7.5%)	[5.5–9.9]	11 (5.4%)	[2.9–9.1]	0.306
	n.r.	166 (95.4%)	[91.5–97.8]	531 (92.5%)	[90.1–94.5]	193 (94.6%)	[90.9–97.1]	
Rapid breathing/tachypnea	Positive	3 (1.7%)	[0.5–4.5]	26 (4.5%)	[3.1–6.5]	10 (4.9%)	[2.5–8.5]	0.212
	n.r.	171 (98.3%)	[95.5–99.5]	548 (95.5%)	[93.5–96.9]	194 (95.1%)	[91.5–97.5]	
Pneumonia	Positive	0 (0%)	[0–0]	5 (0.9%)	[0.3–1.9]	0 (0%)	[0–0]	0.191
	n.r.	174 (100%)	[0–0]	569 (99.1%)	[98.1–99.7]	204 (100%)	[0–0]	
Acute respiratory distress syndrome	Positive	0 (0%)	[0–0]	2 (0.3%)	[0.1–1.1]	0 (0%)	[0–0]	0.517
	n.r.	174 (100%)	[0–0]	572 (99.7%)	[98.9–99.9]	204 (100%)	[0–0]	
Respiratory insufficiency/assisted ventilation	Positive	0 (0%)	[0–0]	1 (0.2%)	[0–0.8]	0 (0%)	[0–0]	0.719
	n.r.	174 (100%)	[0–0]	573 (99.8%)	[99.2–100]	204 (100%)	[0–0]	
Oxygen saturation < 94%	Positive	0	[–]	0	[–]	0	[–]	-
	n.r.	174 (100%)	[0–0]	574 (100%)	[0–0]	204 (100%)	[0–0]	

^1^ Significant *p*-values (*p* < 0.05) from Pearson’s chi squared statistics in bold.

## Data Availability

Data are contained within the article. Further inquiries can be directed to the corresponding authors.
